# Inhibition of glutathione peroxidase 4 suppresses gastric cancer peritoneal metastasis via regulation of RCC2 homeostasis

**DOI:** 10.1016/j.redox.2025.103519

**Published:** 2025-01-30

**Authors:** Can Hu, Jingli Xu, Yanqiang Zhang, Ruolan Zhang, Siwei Pan, Jiahui Chen, Yan Wang, Qianyu Zhao, Yuqi Wang, Weiwei Zhu, Mengxuan Cao, Shengjie Zhang, Dan Zu, Zhiyuan Xu, Ji Jing, Xiangdong Cheng

**Affiliations:** a Department of Gastric Surgery, Zhejiang Cancer Hospital, Hangzhou Institute of Medicine (HIM), Chinese Academy of Sciences, Hangzhou, Zhejiang, 310022, China; b Zhejiang Provincial Research Center for Upper Gastrointestinal Tract Cancer, Zhejiang Cancer Hospital, Hangzhou, 310022, China; c Key Laboratory of Prevention, Diagnosis and Therapy of Upper Gastrointestinal Cancer of Zhejiang Province, Hangzhou, 310022, China; d Hangzhou Institute of Medicine (HIM), Chinese Academy of Sciences, Hangzhou, Zhejiang, 310022, China

**Keywords:** Gastric cancer, GPx4, RCC2, Metastasis, ROS

## Abstract

Gastric cancer (GC) is one of the most lethal malignancies due to high metastatic rate, making the identification of new therapeutic targets critical for developing effective anti-GC treatments. Glutathione peroxidase 4 (GPx4), a key regulator of ferroptosis and redox homeostasis, contributes to progression and influences patient survival. However, the molecular mechanism by which GPx4 drives GC progression has not been fully illuminated. In this study, we found that GPx4 was overexpressed and negatively associated with poor prognosis and distant metastasis, as confirmed by single-cell RNA sequencing (scRNA-seq) and validation with retrospective clinical samples. GPx4 knockdown suppressed GC invasion, migration and peritoneal metastasis in *vitro* and in *vivo*. Proteomic analysis revealed that GPx4 expression regulated the Homeostasis of RCC2, an oncogene link to epithelial-mesenchymal transition (EMT). Furthermore, we demonstrated that the reactive oxygen species (ROS) accumulation induced by GPx4 inhibition or knockdown activated aurora A phosphorylation, leading to RCC2 ubiquitination and degradation, thereby suppressing peritoneal metastasis in GC. We also identified that the Thr418 phosphorylation site is crucial for RCC2 ubiquitination at the K377, initiating its degradation in response to ROS. In conclusion, our results indicate that GPx4 acts as an oncogene in GC, and that suppressing GPx4 prevents GC progression and metastasis by promoting ROS-induced RCC2 ubiquitination and degradation.

## Highlights

• High expression of GPx4 is associated with poor prognosis and distant metastasis in GC.
• GPx4 expression enhances GC cells metastasis via regulating of RCC2 homeostasis in vitro and in vivo.
• ROS Promotes Ubiquitin-Mediated Degradation of RCC2.
• Phosphorylation at T418 and ubiquitination at K377 are crucial for regulating RCC2 degradation in response to ROS.
• ROS accumulation, resulting from either targeting or knocking down GPx4, suppresses peritoneal metastasis by promoting RCC2 ubiquitination through Aurora A.

## Graphical abstract

**Figure:**
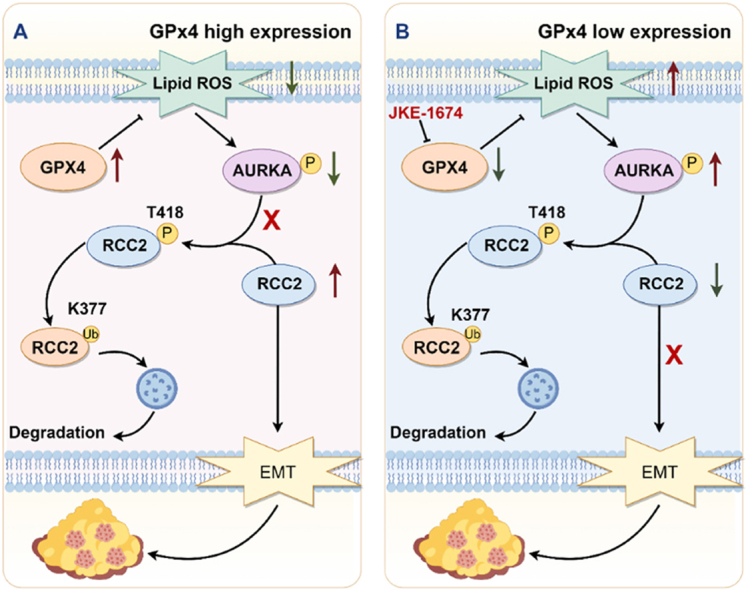

## 1 Introduction

Gastric cancer (GC) is one of the most prevalent malignant tumors globally and poses a serious threat to human health [1]. Metastasis is the primary cause of cancer-related mortality in GC patients, with peritoneum serving as a favorable site for GC cells to grow and form metastatic lesions [2,3]. Peritoneal metastasis in GC is associated with poor prognosis and high mortality [2,4,5]. Despite this, the mechanisms underlying the progression and peritoneal metastasis in GC remain unclear, and investigating these mechanisms could offer new insights in treating these patients.

Glutathione peroxidase 4 (GPx4) is a key regulator involved in multiple physiological and pathological processes [6]. As a member of the peroxidase enzyme family, GPx4 plays a crucial role in eliminating excess reactive oxygen species (ROS) to maintain cellular redox homeostasis [7]. Additionally, GPx4 is a critical regulator of ferroptosis, a newly defined form of regulated cell death primarily caused by iron-mediated lipid peroxidation due to imbalances in cellular antioxidants driven by abnormal cysteine metabolism [8,9]. Hence, targeting GPx4 to induce ferroptosis in cancer cells presents a promising strategy for anticancer drug development [10]. In addition, GPx4 also influencing metastasis in cancer. It has been found to be overexpressed in various cancers, including liver cancer [11], lung cancer [12,13], melanoma [14] and glioma [15], suggesting a potential role in carcinogenesis. Our previous studies have shown that GPx4 expression progressively increases with the advancement of GC [16]. However, the mechanism by which GPx4 regulates distant metastasis in GC remains unclear.

Regulator of chromosome condensation 2 (RCC2), a member of the RCC1 superfamily, is predicted to form a seven-bladed beta-propeller protein that is essential for the chromosomal passenger complex function. It has been identified as an oncogene in various tumors [[17], [18], [19]]. RCC2 regulates cell proliferation by localizing the chromosomal passenger complex (CPC) and Haspin kinase activity to centromeres [20]. Pang et al. [21]. demonstrated that RCC2 overexpression promotes lung adenocarcinoma metastasis by inducing epithelial-mesenchymal transition (EMT) through MAPK-JNK signaling activation. However, RCC2 loss promotes random migration and leads to a failure to maintain natural tumor boundaries in colorectal cancer [22].

This study provides the first evidence that the role of GPx4 in peritoneal metastasis. Mechanistic investigations reveal that targeting GPx4 suppresses peritoneal metastasis by inducing the accumulation of ROS, which in turn promotes RCC2 phosphorylation, ubiquitination and degradation through the activation of Aurora A. In summary, these findings uncover a previously unknown role of the GPx4-RCC2 signaling axis in the progression and metastasis of GC, identifying GPx4 as a potential therapeutic target for GC patients.

## 2 Materials and methods

### 2.1 Human tissue samples

A total of 14 samples were collected from 5 patients with stage-IV GC treated at Zhejiang Cancer Hospital for single-cell RNA sequencing (scRNA-seq). These included 4 adjacent normal tissues, 5 primary tumor tissues, and 5 ascites fluid samples. This study received approval from the Ethics Committee of Zhejiang Cancer Hospital (No.IRB-2020-109). Additionally, 458 GC tissues and 358 paired adjacent normal tissues were obtained from 458 patients who underwent gastrectomy at Zhejiang Cancer Hospital between 2007 and 2017 for immunohistochemical verification. Demographic information and clinicopathological characteristics, such as age, sex, T stage (which reflects the depth of tumor infiltration), N stage (which reflects tumor lymph node metastasis), M stage (which reflects distant metastasis), TNM stage (which refers to the eighth edition of the AJCC staging standard), HER2 status, and PD-L1 status, were collected to analyze their relationship with GPx4 expression (Supplementary Table 1). This study was approved by the Ethics Committee of Zhejiang Cancer Hospital (No.IRB-2023-960). All patients provided written informed consent to participate. This study conformed to the principles outlined in the Declaration of Helsinki (1964 and later version).

### 2.2 Cell lines and regents

Human GC cell lines (AGS, GCIY, MKN-45, MKN-1, NUGC-4, HGC-27, BGC823, MKN-74) were obtained from Shanghai Bioleaf Biotech Co., Ltd. (Shanghai, China). The human gastric epithelial cell line GES-1 was obtained from the Cell Bank of the Chinese Academy of Science (Shanghai, China). All cell lines were recently authenticated by short tandem repeat authentication and tested for mycoplasma contamination.

### 2.3 Immunohistochemistry (IHC)

Primary GC tissues and adjacent noncancerous tissues from 458 patients who underwent gastrectomy at Zhejiang Cancer Hospital between 2007 and 2017 were collected. Tissue microarrays (TMAs) were constructed, including 458 GC tissues and 358 noncancerous tissues, followed by IHC staining and analysis. IHC staining was conducted using antibodies against GPx4 (#ab125066, Abcam), RCC2 (#5104, Cell Signaling technology) according to standard protocols to accesses protein expression levels [16]. Protein expression was evaluated using the H-score system. The formula for the H‐score was as follows: H‐score = ∑ (IS × AP), where IS represents staining intensity (0, no staining; 1, weak; 2, moderate; 3, strong) and AP represents the percentage of positively stained tumor cells (0, <5 %; 1, 5–25 %; 2, 26–50 %; 3, 51–75 %; 4, 76–100 %), resulting in an H-score range of 0–12. To determine the average staining intensity within a tumor sample, multiple regions were analyzed, with at least 100 tumor cells evaluated. Scoring was independently performed by two experienced pathologists who were blinded to clinical outcomes.

### 2.4 Immunofluorescence staining

GC cells were fixed with 4 % paraformaldehyde, permeabilized with 0.1 % Triton X-100, and blocked with 5 % BSA. Cells were incubated with a GPX4 primary antibody overnight at 4 °C, followed by a fluorescent secondary antibody for 1 h. Nuclei were stained with DAPI, and images were captured using a fluorescence microscope.

### 2.5 Generation of concentrated lentiviral vector and infection

Lentiviral vectors containing shRNA targeting GPx4, GPx4 overexpression constructs, negative control shRNA, RCC2 overexpression, or RCC2 mutation were synthesized by GeneChem Biotechnology Company (Shanghai, China). The sequences of 21-nucleotide shRNAs targeting GPx4 were GTGGATGAAGATCCAACCCAA and TTGGGTTGGATCTTCATCCAC, GTGAGGCAAGACCGAAGTAAA and TTTACTTCGGTCTTGCCTCAC. GC cells were transfected with lentivirus according to the manufacturer's instructions. After 72 h, stable cell lines were selected using 1 μg/ml puromycin. Transfection efficiency was confirmed by Western blotting.

### 2.6 RNA isolation and quantitative RT-qPCR

Total RNA was extracted using the RNA-Quick Purification Kit (Yishan Biotech, Shanghai, China) and reverse transcribed with the ReverTra Ace qPCR RT kit (Yishan Biotech, Shanghai, China). Subsequently, Quantitative PCR (qPCR) was subsequently performed using SYBR Green reagent (Yishan Biotech, Shanghai, China) on a CFX96 Touch Real-Time PCR Detection System (Bio-Rad). Relative gene expression was calculated using the 2−ΔΔCq method. The primers used in this study were as follows: GAPDH forward, 5′- CATGTTCGTCATGGGTGTGAA-3′ and reverse, 5′-CGCATGGACTGTGGTCATGAG-3′, GPx4 forward, 5′-ATGGTTAACCTGGACAAGTACC-3′ and reverse, 5′-GACGAGCTGAGTGTAGTTTACT-3′, RCC2 forward, 5′-GGTCTTAGCCACGAAGTGATTG-3′ and reverse, 5′-TCGTACTCTATCCGCTGTGCC-3′.

### 2.7 Western blotting analysis

Cells were lysed in 1 × sodium dodecyl sulfate (SDS) lysis buffer, and total protein was quantitated. Proteins were separated by SDS‒PAGE and transferred to PVDF membrane (Millipore, MA, USA). The target proteins were detected using primary antibodies against GPx4 (Abcam, ab1250668, 1/1000), RCC2 (Cell Signaling technology, #5104, 1/1000), phosphor-ATM S1981 (Abcam, ab81292, 1/1000), ATM (Abcam, ab32420, 1/1000), phosphor-RCC2 T418 (generated by HUBIO), p-Aurora A (CST, 2914S, 1/1000), Flag (Sigma, F1804-200UG, 1/1000), HA (CST, 3724S, 1/1000), N-cad (Abcam, ab76011, 1/1000), E-cad (Abcam, ab40772, 1/1000), and GAPDH (#60004-1-Ig, ProteinTech). Secondary antibodies used included anti-mouse IgG (#926–6807, Invitrogen) and anti-rabbit IgG (#926–68070, Invitrogen). Protein bands were visualized using enhanced chemiluminescence (ECL; Fdbio Science, Hangzhou, China), and band intensity was quantified using ImageJ software.

### 2.8 Sample preparation and intervention

For the preparation of gastric cancer tissues and normal frozen samples, sterile forceps were utilized to finely mince the tissues. Following this, RIPA lysis buffer was added, and the samples were rapidly frozen using liquid nitrogen before being homogenized to obtain the final protein extracts, which were subsequently analyzed using Western blotting.

To investigate the effects of the GPX4 inhibitor JEK-1674 (MCE) on GC cell lines MKN1 and NUGC4, cells were treated with varying concentrations of JEK-1674 (0.5, 0.75, 1.0, and 2.0 μM) for 24 h. Post-treatment, cells were harvested for further Western blot analysis and GPx activity assays.

In experiments designed to simulate reactive oxygen species (ROS) and lipid peroxidation, MKN1 cells were treated with H_2_O_2_ (0, 0.4, 0.8, 1.2, and 2.0 mM) or 4-HNE (0, 16, 32, 48, and 64 μM) for 6 h. Similarly, NUGC4 cells were exposed to H_2_O_2_ (0, 0.6, 0.8, 1.6, and 3.0 mM) or 4-HNE (0, 32, 64, 96, and 128 μM) for the same duration.

To explore the kinases responsible for RCC2 phosphorylation modifications, MKN1 and NUGC4 cells were co-treated with JEK-1674 (4 μM) and Alisertib (200 nM) for 48 h. Following treatment, Western blot analysis and Transwell assays were performed for validation. In experiments investigating the role of GPX4 in regulating RCC2 expression, MKN1 and NUGC4 cells were co-treated with JEK-1674 (4 μM) and NAC (20 mM) for 24 h. Additionally, MKN1 and NUGC4 cells with GPX4 knockdown were treated with JEK-1674 (4 μM) for comparison. Following treatment, Western blot analysis was conducted for validation.

### 2.9 Transwell migration and invasion experiments

Migration and invasion assays were performed using the transwell chambers without (migration) and with (invasion) growth factor–reduced Matrigel (Corning). Briefly, 5 × 10^4^ cells in FBS-free medium were plated in the top chamber, while growth medium containing 10 % FBS was used as a chemoattractant in the lower chamber. After the specified indication periods, migrated and invaded cells were fixed and stained with 0.1 % crystal violet. Images were captured using a Nikon Digital Sight DS-L1 camera.

### 2.10 Quantitative proteome analysis

Cells with stable control and shGPx4 were harvested and sent to GeneChem Biotechnology Company (Shanghai, China) for the creation of a quantitative proteome library and subsequent sequencing. This involved a series of processes including protein extraction, quantification, enzymatic digestion, and mass spectrometry analysis (MS). The MS data were analyzed using MaxQuant software (version 1.6.17.0) and searched against a relevant the database. The acceptable threshold for the global false discovery rate (FDR) for both peptide and protein identification were set to 0.01. Protein levels were calculated by normalized spectral protein intensity (LFQ intensity). Proteins exhibiting a fold change >1.5 and *P*-value (Student's t-test) <0.05 were identified as significantly differentially expressed [23].

### 2.11 Detection of LipidROS levels

The level of intracellular ROS levels was determined using the C11-BODIPY 581/591 Kit (Invitrogen, #D-3861). Cells were stained with 2 μmol/L C11‐BODIPY^581/591^ probe following the manufacturer's instructions. After a 30 min incubation period at 37 °C in darkness, the fluorescence of C11‐BODIPY^581/591^ was performed using flow cytometry and the data was analyzed with the FlowJo V.10 software.

### 2.12 GPx activity assay

According to the manufacturer's instructions, Glutathione peroxidase (GPx) activity was detected using the Glutathione Peroxidase Assay Kit (Abcam, Cat No. ab102530).

### 2.13 Coimmunoprecipitation

To explore the degradation pathways of RCC2, MKN1 cells were treated with 0.4 mM H2O2 for 0, 1, 3, and 6 h. Another group of cells was pre-treated with 5 μM MG132 or 100 nM BafA1 for 2 h, followed by co-treatment with 0.4 mM H_2_O_2_ for the same time points. Cells were then collected for Western blot analysis. Additionally, MKN1 cells transfected with Flag-RCC2 and HA-ub were harvested at 0 and 6 h post-H2O2 treatment (with or without MG132) for subsequent co-immunoprecipitation (Co-IP) using anti-Flag and anti-HA antibodies.

To identify modification sites on RCC2, MKN1 and NUGC4 cells were transfected with Flag-RCC2 wild-type (wt) and various Flag-RCC2 mutant constructs. After a 6-h treatment with 0.4 mM H_2_O_2_, cells were collected for Western blot analysis. Concurrently, cells were co-transfected with HA-ub plasmid alongside Flag-RCC2 wt and the corresponding mutants. Following H_2_O_2_ treatment, cells were harvested for Co-IP with anti-Flag antibodies, followed by Western blot detection.

For the purpose of coimmunoprecipitating endogenous proteins, cellular lysates were incubated with primary antibodies or control IgG and agitated in a rotatory incubator at 4°Covernight. This was followed by the addition of protein A/G magnetic beads (Bimake) and a further 2 h incubation. The immunoprecipitants were washed 3 times with lysis buffer before being subjected to immunoblotting for analysis.

### 2.14 Establishment of peritoneal metastasis model in mice

To evaluate the role of GPX4 in peritoneal metastasis, MKN-45 cells, at total of 6 × 10^6^ suspended in 200 μL saline solution, were administered intraperitoneally to male nude mice aged five to six weeks [24]. The mice were randomly and blindly divided to three groups: control, shGPx4#1, and shGPx4#2 groups (*n* = 5/each group). To validate that GPX4 regulates tumor growth through ROS, MKN1 cells were modeled using the same method and divided into the following groups: control, shGPX4, and shGPX4 receiving NAC (100 mg/kg, intraperitoneal injection, twice a week). For the functional validation of GPX4-related sites, MKN45 cells from the vector control group (Vector group), the wild-type RCC2 overexpression group (RCC2^WT^ group), and the T418 mutant RCC2 overexpression group (RCC2^T418A^) were also inoculated into the abdominal cavities of mice. Each group was then randomly divided into two subgroups, which received either saline or JEK-1674 (10 mg/kg) via intraperitoneal injection every two days for treatment (*n* = 5/each group). Throughout the study, the body weight, living status, and tumor size of the mice were recorded. At the end of the 28-day post-injection period, the mice were sacrificed, and a small shallow section was made to expose the abdominal cavity. The number of macroscopic nodules was then recorded, with the experimenter remaining blinded to the group assignments. The protocol was approved by the Committee on the Ethics of Animal Experiments of Zhejiang Chinese Medical University.

### 2.15 In vivo luminescence imaging

Prior to imaging, the mice were rendered unconscious using anesthesia. Bluminescence was measured 5 min post-intraperitoneal injection of D-luciferin sodium salt at a dosage of 150 mg/kg, utilizing the in vivo imaging system (IVIS) Lumina LT (Caliper Life Sciences, USA). The luciferase signal, indicative of the extent of peritoneal metastasis, was quantified with the IVIS system. The living Image software Ver. 4.3 (Caliper Life Sciences, USA) was employed to process the images and acquire the data.

### 2.16 Statistical analysis

Statistical evaluations were conducted utilizing GraphPad Prism software, version 6.0. Parametric assessments (Student's t-test or one-way ANOVA) applied for normally distributed data, and nonparametric tests used for data that did not meet these criteria. Survival curves were generated using the Kaplan‒Meier method. Count data are presented as the rate or composition ratio using the chi-square test. Data are presented as the mean ± SEM, with statistical significance is adjudicated at a *P* value of less than 0.05, with specific significance thresholds marked as ∗*P* < 0.05, ∗∗*P* < 0.01, ∗∗∗*P* < 0.001, and ∗∗∗∗*P* < 0.0001; 'ns' denotes not significant.

## 3 Results

### 3.1 High expression of GPx4 is associated with poor prognosis and distant metastasis in GC

**Fig. 1:**
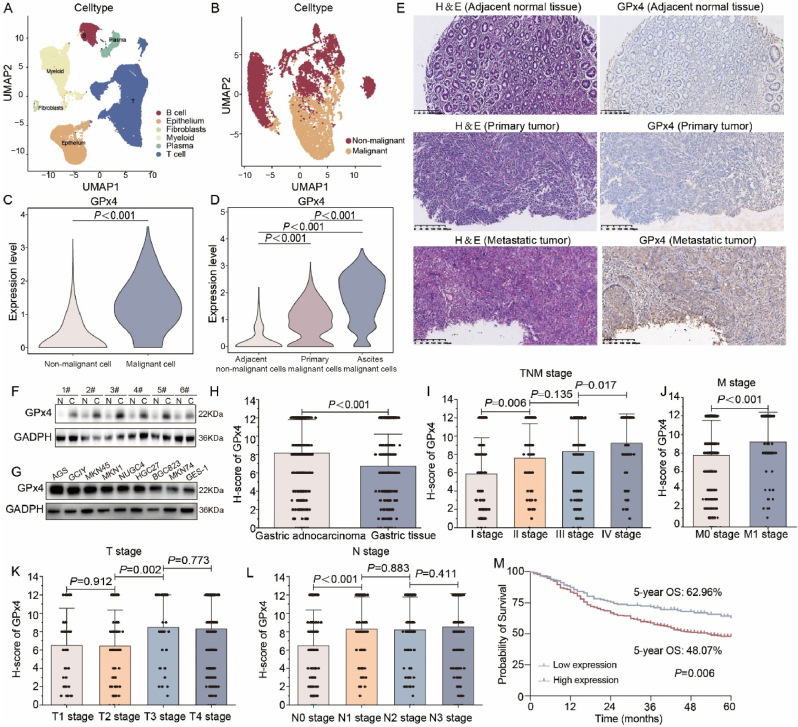
**GPx4 was associated with the progression and prognosis of GC**. **A**. UMAP plot illustrates the segregation of predominant cell types. Different colors correspond to distinct cell types. **B**. A UMAP plot highlighting the distribution patterns of malignant cells and non-malignant cells in epithelium. **C**. The panel displays the expression levels of GPx4 in malignant cells and non-malignant cells in epithelium. **D**. The panel displays the expression levels of GPx4 in normal epithelial cells from adjacent normal tissues, and in malignant cells derived from primary tumors and ascites. **E**. Representative images of H&E and immunohistochemistry images demonstrate GPx4 protein levels in adjacent normal tissues, primary tumor and paired metastases sites. **F**. Western blot analysis compares GPx4 expression between GC tissues and their adjacent normal tissues (N = 6). **G**. Western blot analysis of GPx4 expression in normal gastric epithelial cells (GES-1) and a panel of gastric cancer cell lines (MKN-45, MKN-74, NUGC-4, MKN-1, NUGC-3, AGS). **H**. Histogram showing the H-score of GPx4 expression in 458 gastric adenocarcinoma samples and 358 matched normal gastric tissues. **I**. H-score of GPx4 expression in different TNM stages. **J**. H-score of GPx4 expression in various M stages. **K**. H-score of GPx4 expression in different T stages. **L**. H-score of GPx4 expression in different N stages. **M**. Correlation analysis between GPx4 expression levels and 5-year overall survival (OS) in GC.

To investigate transcriptomic changes between primary and metastatic tumors, we performed single-cell RNA sequencing (scRNA-seq) on GC patients with peritoneal metastasis. After quality filtering, single-cell transcriptomic data were obtained for subsequent analysis [25,26]. Following data preprocessing and principal component analysis (PCA), we applied graph-based uniform manifold approximation and projection (UMAP) to categorize the cells (Fig. 1A–B). Notably, GPx4, a key regulator of ferroptosis, showed higher expression in malignant cells compared to non-malignant cells in epithelium (Fig. 1C). Further analysis showed that a progressive increase in expression from normal epithelial cells in adjuvant normal tissues to tumor cells in primary tumors and ascites, indicating its significant role in GC development and metastasis (Fig. 1D). To investigate GPx4 protein expression during GC progression, we analyzed its expression across various stages of GC. Immunohistochemical analysis revealed that GPx4 was overexpressed in distant metastasis compared with primary tumor and adjacent normal tissues (Fig. 1E). Western blot analysis further confirmed that GPx4 was highly expressed in GC tissues compared to paired normal gastric tissue (Fig. 1F–G). Additionally, GPx4 expression was assessed in GC tissue microarrays (TMAs) using immunohistochemistry (IHC), which localized GPx4 predominantly in the cytoplasm (Supplementary Fig. 1A). The intensity of GPx4 staining was significantly stronger in GC tissues compared to paired normal gastric tissues, with a mean H-score of 8.16 in GC tissues versus 6.75 in normal gastric tissues (Fig. 1H). Based on the H-score distribution, an H-score <8. Was defined as low GPx4 expression, and H-score≥8.0 as high GPx4 expression. In GC tissues, 304 patients (66.38 %) exhibited high GPx4 expression, while 154 (33.62 %) had showed low expression. In contrast, in normal gastric tissues, 167 patients (46.65 %) showed high GPx4 expression, and 191 (53.35 %) showed low GPx4 expression (*P* < 0.001, as shown in Supplementary Table 2).

We then examined the relationship between GPx4 expression and clinicopathological characteristics in GC patients. High GPx4 expression was significantly correlated with advanced N stage (*P* = 0.038), M stage (*P* = 0.023), and TNM stage (*P* = 0.023), but not with other factors such as age, gender, tumor location, Lauren type, Borrmann type, differentiated degree, T stage, HER2 status, or PD-L1 status (Supplementary Table 3). Univariate analysis revealed that GPx4 expression (*P* = 0.007), tumor location (*P* < 0.001), T stage (*P* = 0.001), N stage (*P* = 0.001), M stage (*P* < 0.001), and TNM stage (*P* < 0.001) as prognostic risk factors for GC patients. Furthermore, multivariate Cox regression analysis indicated that GPx4 expression (*P* = 0.030), T stage (*P* = 0.031), N stage (*P* = 0.034), and M stage (*P* < 0.001) are independent prognostic risk factors (Fig. 1I–L, Supplementary Table 4). Importantly, a significant difference in 5-year overall survival (OS) was observed between patients with high versus low GPx4 (48.07 % vs. 62.96 %, *P* = 0.006, Fig. 1M). These findings suggest that GPx4 expression is closely associated with metastasis and poor prognosis in GC.

### 3.2 GPx4 expression enhances GC cells metastasis in *vitro* and in *vivo*

**Fig. 2:**
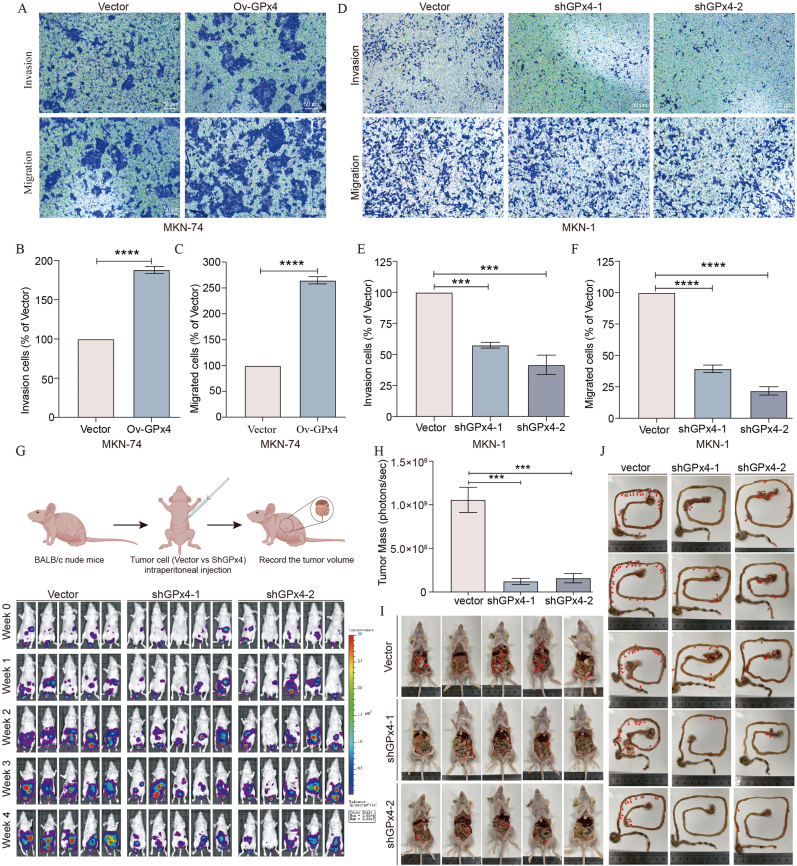
**GPx4 expression enhances GC metastasis in *vitro* and in *vivo***. **A**. Transwell assay results post-GPx4 overexpression (scale bar, 50 μm). **B–C**. Quantitative analysis of transwell assay data post-GPx4 overexpression. **D**. Transwell assay results post-GPx4 knockdown (scale bar, 50 μm). **E-F**. Quantitative analysis of transwell migration assay data post-GPx4 knockdown. **G**. Nude mice were intraperitoneally injected with GC cells stably transfected with GPx4 shRNA or an empty vector. The mice were randomly divided to control, shGPx4-1, and shGPx4-2 groups as detailed in the Methods. Luciferase signals were detected in the mice using an IVIS imaging system, and representative images were captured. **H**. Mean tumor mass (as measured by detected photons/sec) in the control, shGPx4-1, and shGPx4-2 groups of mice. **I-J**. Assessment of peritoneal and intestinal metastasis in the control, shGPx4-1, and shGPx4-2 groups of mice.

Clinical data suggest that GPx4 expression may play a crucial role in GC metastasis. To investigate this, we generated stable GPx4 knockdown MKN-1, NUGC-4 and MKN-45 GC cell lines using GPx4-specific shRNA, as well as stable GPx4-overexpressing MKN-74 GC cells (Supplementary Figs. 1B–1C). Transwell assays demonstrated a significantly decrease in migration and invasion abilities in GPx4 knockdown MKN-1 cells, while these abilities were enhanced in GPx4-overexpressing MKN-74 cells (Fig. 2A–F). In addition, we found that the ability of invasion and migration could be rescued after overexpressing GPx4 in MKN-1 shGPx4 GC cells (Supplementary Figs. 1D–1E). To further assess the impact of GPx4 on metastasis in vivo, we established a peritoneal metastasis model. GPx4 knockdown MKN-45 GC cells or control MKN-45 GC cells were injected into the abdominal cavities of mice, followed by intraperitoneal administration of fluorescein substrate (150 mg/kg) for in vivo imaging using a Xenogen IVIS 200 imaging system once a week (Fig. 2G). Analysis with LT Living Image 4.3 Software confirmed that GPx4 knockdown significantly suppressed GC metastasis. Additionally, the number of metastatic nodules and weight of tumor in the peritoneal cavity was significantly decreased in the shGPx4-1 and shGPx4-2 groups compared to the control group (Fig. 2H–J, Supplementary Figs. 1F–1G).

### 3.3 Proteomic analysis reveals RCC2 downregulation following GPx4 knockdown

**Fig. 3:**
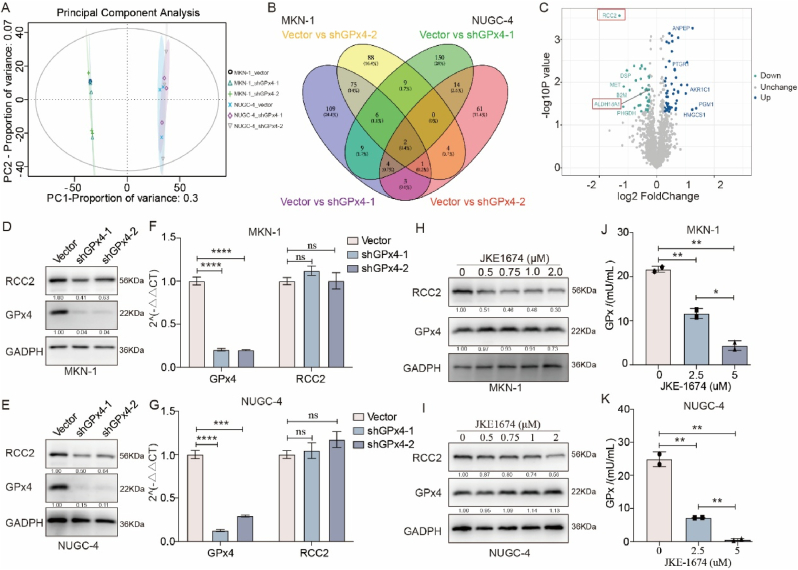
**Suppression of GPx4 leads to reduced RCC2 expression**. **A**. PCA analysis of the mass spectrometry data. **B**. Venn diagram illustrating the overlap of differentially expressed proteins in MKN-1 and NUGC-4 GC cells following GPx4 knockdown. **C**. Volcano plot representing the proteins with significant expression changes in MKN-1 GC cells transfected with shGPx4-1. **D-E**. Western blot analysis of RCC2 expression in MKN-1 and NUGC-4 GC cells after transfection with GPx4-shRNA and scrambled-shRNA. **F-G**. RT-qPCR analysis of GPx4 and RCC2 mRNA expression in MKN-1 and NUGC-4 GC cells following transfection with GPx4-shRNA and scrambled-shRNA. **H–I**. Western blot analysis of RCC2 expression in MKN-1 and NUGC-4 GC cells treated with various concentrations of JEK-1674 (0, 0.5, 0.75, 1.0, 2.0 μM) for 24 h. **J-K**. Assessment of GPxs activity in MKN-1 and NUGC-4 GC cells after treatment with JEK-1674 (0, 2.5, 5.0 μM) for 24 h.

To investigate the molecular mechanisms by which GPx4 promotes GC cell metastasis, we performed quantitative proteome analysis on MKN-1 and NUGC-4 GC cells, as well as their respective GPx4 knockdown counterparts. PCA indicated a shift in both principal component 1 (PC1) and PC2 (Fig. 3A). In total, 4, 528 proteins were identified across all samples. Comparative analysis of differentially expressed proteins between GPx4 knockdown and control cells in both MKN-1 and NUGC-4 GC cells revealed significant changes in RCC2 and ALDH18A1 expression following GPx4 knockdown (Fig. 3B and C). RCC2 and ALDH18A1 high expression have been confirmed associated with tumor progression [21,27,28]. Next, we focused on RCC2 expression, as RCC2 levels decreased, while ALDH18A1 expression increased after GPx4 knockdown. Western blot analysis confirmed that GPx4 knockdown significantly reduced RCC2 expression in both MKN-1 and NUGC-4 GC cells (Fig. 3D and E). However, quantitative PCR analysis revealed no significant change in RCC2 RNA levels following GPx4 knockdown (Fig. 3F and G), suggesting that GPx4 regulates RCC2 expression at the protein level rather than through transcriptional changes. Additionally, treatment with the GPx4 inhibitor JKE-1674 for 24 h led to dose-dependent reduction in RCC2 protein levels (Fig. 3H–I) and GPxs activity (Fig. 3J–K) in these cells.

### 3.4 ROS Promotes Ubiquitin-Mediated Degradation of RCC2

**Fig. 4:**
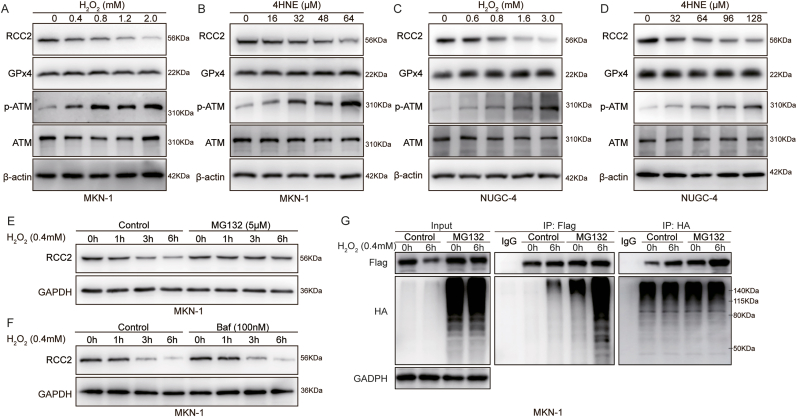
**ROS accumulation promotes RCC2 degradation via proteasomal pathway. A-B.** Western blot analysis of RCC2, GPx4, phosphorylated ATM (*p*-ATM) and total-ATM expression in MKN-1 GC cells following treatment with varying concentrations of H_2_O_2_ (0, 0,4, 0,8, 1.2, 2.0 mM) or 4HNE (0, 16, 32, 48, 64 μM) for 6 h. **C-D**. Western blot analysis of RCC2, GPx4, *p*-ATM and ATM expression in NUGC-4 GC cells after treatment with different concentrations of H_2_O_2_ (0, 0,6, 0,8, 1.6, 3.0 mM) or 4HNE (0, 32, 64, 96, 128 μM) for 6 h. **E**. MKN-1 GC cells were pre-treated with the proteasome inhibitor MG-132 (5 μM) for 2 h, followed by co-treatment with H_2_O_2_ (0.4 mM) for an additional 6 h. RCC2 expression was then detected by Western blot. **F**. MKN-1 GC cells were pre-treated with Bafilomycin A1 (100 nM) for 2 h, after which the cells were co-treated with H_2_O_2_ (0.4 mM) for 6 other hours. RCC2 protein expression was detected by Western blot. **G**. MKN-1 cells co-transfected with Flag-RCC2 and HA-tagged Ubiquitin (HA-Ub) plasmids were pre-treated with MG-132 (5 μM) for 2 h, followed by treatment with or without H_2_O_2_ (0.4 mM) for an additional 6 h, respectively. Protein samples were subjected to immunoprecipitation using anti-Flag antibody, followed by Western blot analysis with anti-HA antibody, and vice versa.

GPx4 plays a crucial role in maintaining cellular viability by scavenging ROS. Elevated ROS levels are known to contribute to tumorigenesis, cancer progression and metastasis by promoting and sustaining tumorigenic signaling pathways, leading to increased tumor cell proliferation, survival, autophagy, and metastasis [29]. Our results confirmed that lipid peroxidation levels increased in a dose-dependent manner following treatment with GPx4 inhibitor JKE-1674 (Supplementary Fig. 2). N-acetyl-l-cysteamine (NAC, a ROS scavenger) could reduce the effect of GPx4 knockdown in peritoneal metastasis (Supplementary Fig. 3). This led us to hypothesize that GPx4 inhibition might downregulate RCC2 expression by modulating ROS levels. To test this, we treated MKN-1 and NUGC-4 GC cells with exogenous ROS (H_2_O_2_) or lipid ROS (4-HNE), using phosphorylated ATM (*p*-ATM) protein as positive control [30]. The results demonstrated that both H_2_O_2_ and 4-HNE significantly reduced RCC2 expression in a dose-dependent manner (Fig. 4A and D).

Next, we explored the mechanism by which ROS regulates RCC2 expression. Pre-treatment with the proteasome inhibitor MG-132 (5 μM) significantly inhibited the H_2_O_2_ (0.4 μM) induced downregulation of RCC2, whereas pre-treatment with the autophagy inhibitor Bafilomycin A1 (100 nM) did not have a significant effect (Fig. 4E and F). This suggests that ROS-mediated downregulation of RCC2 is likely linked to proteasomal degradation. To further confirm that ROS induces RCC2 degradation via the ubiquitin-proteasome system, we performed immunoprecipitation using MKN-1 cells transfected with exogenous RCC2 and ubiquitin (Ub) plasmids. The results showed that H_2_O_2_ treatment significantly enhanced RCC2 ubiquitination in the presence of MG-132 (Fig. 4G), which subsequently led to its degradation by the proteasome system. These results suggest that GPx4 inhibition may regulate the ubiquitin-mediated degradation of RCC2 via ROS accumulation.

### 3.5 ROS promote RCC2 ubiquitination at K377

**Fig. 5:**
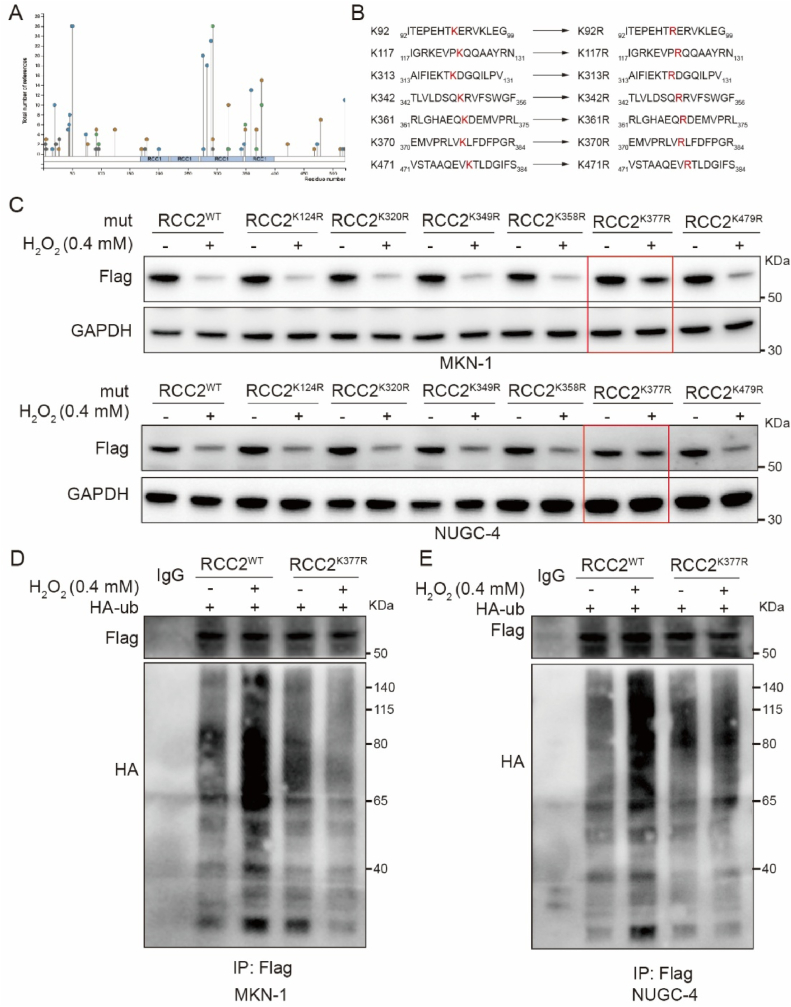
**ROS accumulation promotes RCC2 ubiquitination at K377. A.** Analysis using the PhosphoSite database (https://phosphosite.org) identified potential ubiquitination sites on RCC2 under medium stringency conditions. **B**. Site-directed mutagenesis was performed to convert lysine residues 92, 124, 320, 349, 368, 377, or 479 of RCC2 to arginine (K92R, K124R, K320R, K349R, K368R, K377R, or K479R). **C.** Western blot analysis of RCC2 expression in MKN-1 and NUGC-4 GC cells transfected with specific site-directed mutants (K124R, K320R, K349R, K368R, K377R, or K479R) following a 6-h treatment with H_2_O_2_ (0.4 mM). **D-E.** MKN-1 and NUGC-4 cells co-transfected with Flag-RCC2 (RCC2^WT^ or RCC2^K377R^) and HA-Ub plasmids were treated with or without H_2_O_2_ (0.4 mM) for 6 h. Ubiquitination were analyzed by immunoprecipitation with an anti-Flag antibody, followed by Western blot with anti-HA antibody.

Prediction of ubiquitination sites on RCC2 using PhosphoSite (https://phosphosite.org) identified K92, K124, K320, K349, K368, K377, and K479 as potential ubiquitination sites of RCC2 (Fig. 5A). To investigate the role of these sites, we performed site-directed mutagenesis, substituting lysine residues with arginine at positions K92, K124, K320, K349, K368, K377, and K479 generating the mutants RCC2^K92R^, RCC2^K124R^, RCC2^K320R^, RCC2^K349R^, RCC2^K368R^, RCC2^K377R^, and RCC2^K479R^ (Fig. 5B). The results showed that RCC2 degradation was not obvious in RCC2 ^K377R^ MKN-1 and NUGC-4 cells after H_2_O_2_ treatment (Fig. 5C). Additionally, immunoprecipitation experiment using MKN-1 and NUGC-4 cells expressing either RCC2^WT^ or RCC2^K377R^, co-transfected with Flag-RCC2 and HA-Ub plasmids, revealed that wild-type RCC2 underwent ubiquitination following H_2_O_2_ treatment, whereas the K377R–RCC2 did not (Fig. 5D and E). These results suggest that the K377 site is essential for RCC2 ubiquitination in response to ROS.

### 3.6 ROS promote RCC2 ubiquitination by phosphorylating RCC2 at T418

**Fig. 6:**
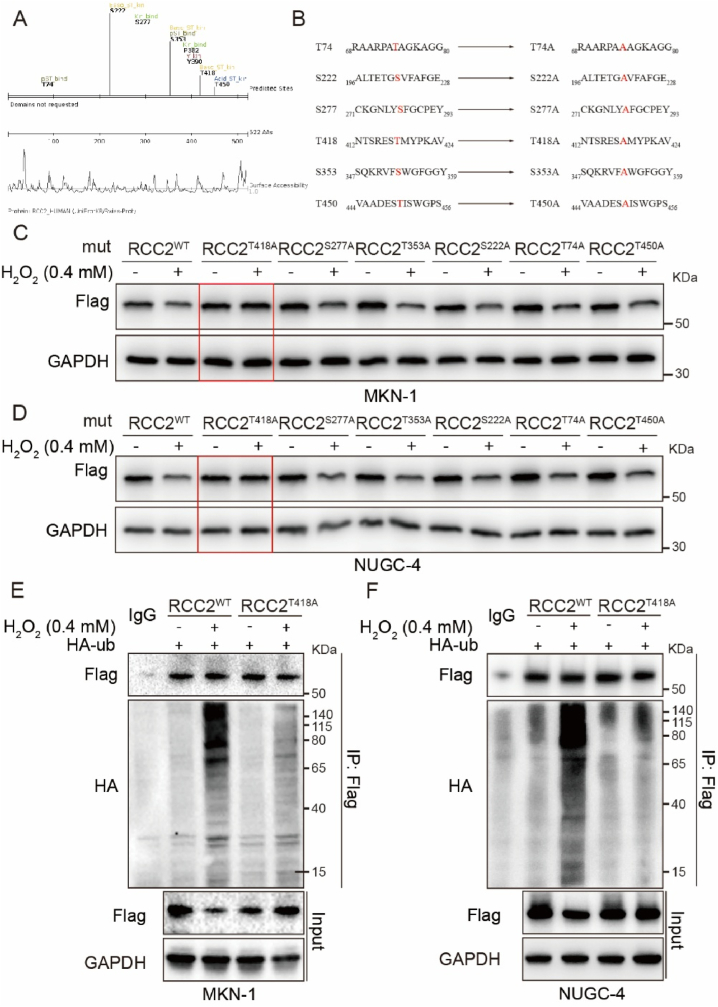
**T418 regulates RCC2 ubiquitination in response to ROS. A.** The SCANSITE database (SCANSITE 4.0, https://scansite4.mit.edu) analysis revealed potential phosphorylation sites on RCC2 under medium stringency conditions. **B**. Site-directed mutagenesis was used to mutate threonine 74, 418, or 450; and serine 222, 277, or 353 of RCC2 to alanine (T74A, T418A, T450A, S222A, S277A, or S353A. **C-D.** Western blot analysis of RCC2 expression in MKN-1 and NUGC-4 GC cells transfected with specific site-directed mutations (WT, T418A, S277A, T535A, T222A, T74A, or T450A) after a 6-h treatment with H_2_O_2_ (0.4 mM). **E-F.** MKN-1 and NUGC-4 cells co-transfected with Flag-RCC2 (RCC2^WT^ or RCC2^T418A^) and HA-Ub plasmids were treated with or without H_2_O_2_ (0.4 mM) for 6 h. Ubiquitination was analyzed by Immunoprecipitation with an anti-Flag antibody, followed by Western blot with an anti-HA antibody; immunoprecipitation with an anti-HA antibody, followed by Western blot with an anti-Flag antibody.

Previous studies have suggested that protein ubiquitination degradation often require prior phosphorylation [31]. Using SCANSITE database (SCANSITE 4.0, https://scansite4.mit.edu), we identified T450, S222, T74, S353, T418, and S277 as potential phosphorylation sites on RCC2 under medium stringency conditions (Fig. 6A). Site-directed mutagenesis was then performed, substituting threonine and serine residues with alanine at positions T74, T418, T450, S222, S277, and S353, generating the mutants RCC2^T74A^, RCC2^T418A^, RCC2^T450A^, RCC2^T222A^, RCC2^S277A^, andRCC2^T353A^) (Fig. 6B). The result showed that RCC2 degradation was not obvious in RCC2^T418A^ MKN-1 and NUGC-4 cells after H_2_O_2_ treatment (Fig. 6C and D). Immunoprecipitation experiments using RCC2^WT^ or RCC2^T418A^ mutant MKN-1 and NUGC-4 cells co-transfected with Flag-RCC2 and HA-Ub plasmids revealed that the exogenous wild-type RCC2 was ubiquitinated, following H_2_O_2_ treatment, while T418A-RCC2 mutant was not. These results findings indicate that phosphorylation RCC2 at T418 is a prerequisite for RCC2 ubiquitination (Fig. 6E and F). A polyclonal antibody generated against the phospho-T418 RCC2 peptide further confirmed that phosphorylation of RCC2 at T418 increased in response to ROS (Supplementary Fig. 4). This suggests that T418 of RCC2 might by a critical phosphorylation site for H_2_O_2_-induced RCC2 degradation.

### 3.7 GPx4 inhibitor suppresses GC metastasis via RCC2 phosphorylation at T418

**Fig. 7:**
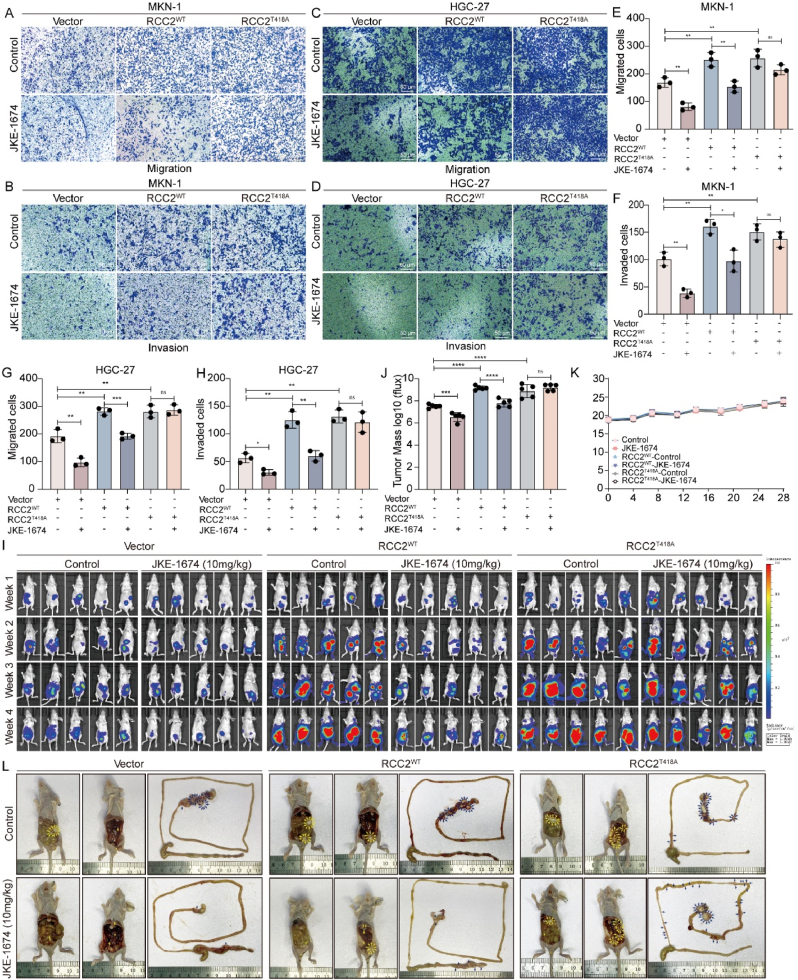
**GPx4 Inhibition impedes GC metastasis via the phosphorylation of RCC2 at T418. A-B.** Transwell migration assays in MKN-1 GC cells transfected with RCC2-WT, RCC2-T418A, or control vector and treated with JKE-1674 (4 μM) or PBS for 48 h (scale bar, 50 μm). **C-D**. Transwell migration assays in HGC-27 GC cells transfected with RCC2-WT, RCC2-T418A, or control vector and treated with JKE-1674 (4 μM) or PBS for 48 h (scale bar, 50 μm). **E-H**. Quantitative analysis of transwell data. **I**. Nude mice were intraperitoneally injected with GC cells stably expressing RCC2-WT, RCC2-T418A, or control vector and randomly assigned to control or JKE-1674 treatment groups as described in the Methods. Luciferase signals were detected using an IVIS imaging system. **J**. Average tumor mass (determined by the detected photons/sec) of the mice across different treatment groups. **K**. Body weight changes in mice from different treatment groups. **L**. Assessment of peritoneal and intestinal metastasis in different treatment groups.

To explore whether GPx4 accelerates GC metastasis by regulating RCC2 phosphorylation at T418, we generated stable RCC2^WT^ and RCC2^T418A^ cell lines. We then assessed the involvement of RCC2 phosphorylation at T418 in GPX4-mediated GC metastasis by treating RCC2^WT^ or RCC2^T418A^ MKN-1 and HGC-27 GC cells with the GPx4 inhibitor JKE1674. The results indicated that the inhibition of GC cell invasion and migration induced by JKE-1674 was partially rescued in RCC2^WT^ GC cells but not in RCC2^T418A^ GC cells in *vitro* (Fig. 7A–H). In addition, RCC2^vector^, RCC2^WT^ or RCC2^T418A^ MKN-45 GC cells were injected into the abdominal cavities of mice, followed by daily intraperitoneal injections of JEK-1674 (2.5 mg/kg) for 3 weeks. The control group received an equivalent volume of solvent. In vivo imaging was conducted weekly using a Xenogen IVIS 200 imaging system after administration of fluorescein substrate (150 mg/kg). Analysis with LT Living Image 4.3 Software confirmed that inhibition of GC peritoneal metastasis induced by JKE-1674 was partially rescued in RCC2^WT^ group but not in RCC2^T418A^ group (Fig. 7J–L, Supplementary Fig. 5).

### 3.8 GPx4 inhibitors promote RCC2 degradation and inhibit GC metastasis by activating aurora A

**Fig. 8:**
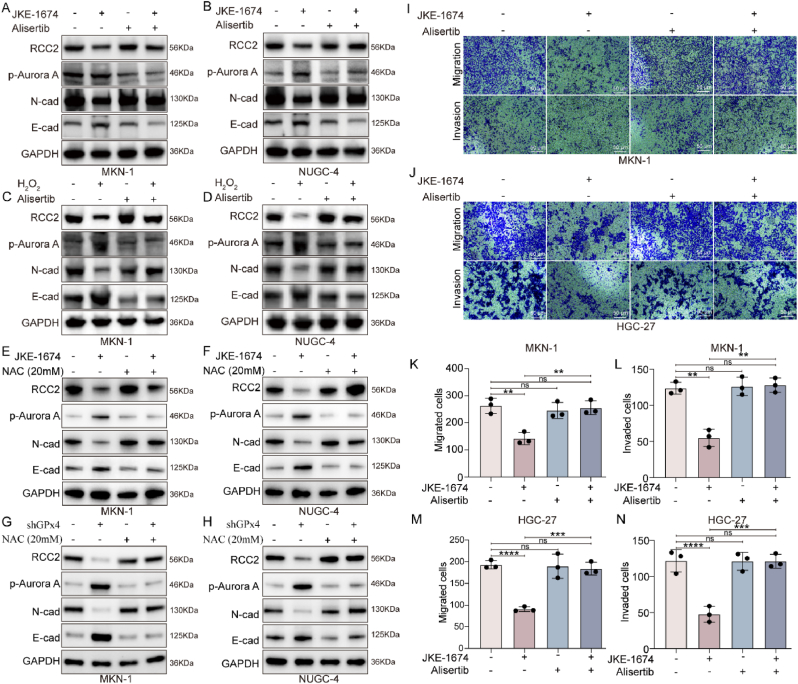
**ROS accumulation from GPx4 inhibition promotes RCC2 degradation and inhibits EMT by activating Aurora A. A-H**. Western blot analysis of RCC2, p-Aurora A, N-cadherin and E-cadherin expression in MKN-1 and HGC-27 GC cells after treatment with JKE-1674 (4 μM), Alisertib (200 nM), NAC (20 mM) or combinations with JKE-1674 and Alisertib or NAC for 24 h. **I-J.** Transwell migration assay in MKN-1 and HGC-27 GC cells after treatment with JKE-1674 (4 μM), Alisertib (200 nM), or combination with JKE-1674 and Alisertib for 48 h (scale bar, 50 μm). **K–N**. Quantitation of the data of transwell assays.

Kinases play a critical role in protein ubiquitination [32]. Kinase prediction analysis (SCANSITE 4.0, https://scansite4.mit.edu) suggested that Aurora A is the kinase responsible for phosphorylating RCC2 at T418 (Supplementary Table 5). Subsequent intervention in MKN-1 and NUGC-4 GC cells treated with JKE-1674 or H_2_O_2_, either alone or in combination with Alisertib (a Aurora A inhibitor) or N-acetyl-l-cysteamine (NAC, a ROS scavenger), showed that JKE-1674 and H_2_O_2_ promoted Aurora A phosphorylation. This phosphorylation, along with the degradation of RCC2, was reversed by Alisertib or NAC. As previous studies have confirmed that RCC2 expression promotes epithelial-mesenchymal transition (EMT) in tumor cells [21], we assessed the switch of E-cadherin and N-cadherin. The results showed that JKE-1674 and H_2_O_2_ promoted EMT, whereas Alisertib or NAC reversed the EMT induced by JKE-1674 or H_2_O_2_ (Fig. 8A–H). Finally, we explored the role of Aurora A in GPx4-mediated GC metastasis. The transwell assays confirmed that Alisertib attenuated the inhibition of migration and invasion in MKN-1 and HGC-27 GC cells treated with JKE-1674 (Fig. 8I–N).

### 3.9 RCC2 expression correlates with GPx4 expression and poor prognosis in GC

**Fig. 9:**
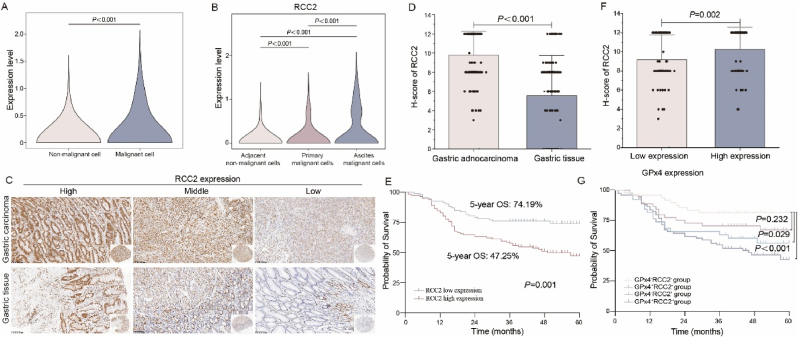
**RCC2 correlates with GPx4 expression and prognosis in GC. A.** Single-cell RNA sequencing analysis of RCC2 expression in malignant cells and non-malignant cells in epithelium. **B**. The panel displays the expression levels of GPx4 in normal epithelial cells from adjacent normal tissues, and in malignant cells derived from primary tumors and ascites. **C**. Representative immunohistochemistry images showing RCC2 expression in gastric carcinoma and paired gastric tissues. **D**. H-score of GPx4 expression in 206 gastric adenocarcinoma samples and corresponding gastric tissues. **E**. Association between RCC2 expression and 5-year OS in GC. **F**. Correlation between GPx4 and RCC2 expression in GC. **G**. Impact of co-expression of GPx4 and RCC2 on 5-year OS in GC.

To access RCC2 expression in GC, scRNA-seq data revealed higher RCC2 expression in malignant cells compared to non-malignant cells in epithelium (Fig. 9A) and a progressive increase in RCC2 expression from normal epithelial cells in adjacent normal tissues to tumor cells in primary tumors and ascites (Fig. 9B). IHC analysis of 206 tissue samples showed that RCC2 was primarily localized in the nucleus (Fig. 9C), with H-score ranging from 0 to 12, and a median score of 8 (Fig. 9D). Consequently, RCC2 expression was significantly elevated in GC compared to adjacent normal tissues (*P* < 0.001). Kaplan-Meier survival analysis indicated that patients with high RCC2 expression had significantly lower 5-year survival rates compared to those with low expression (74.19 % vs 47.25 %, *P* = 0.001) (Fig. 9E).

Further analysis demonstrated that patients with high GPx4 expression had significantly higher RCC2 levels compared to those with low GPx4 expression (*P* = 0.002) (Fig. 9F). Patients were stratified into four groups based on GPx4 and RCC2 expression: low GPx4/low RCC2 (GPx4^−^RCC2^-^), low GPx4/high RCC2 (GPx4^−^RCC2^+^), high GPx4/low RCC2 (GPx4^+^RCC2^-^), and high GPx4/high RCC2 (GPx4^+^RCC2^+^). 5-year OS were lowest in the GPx4^+^RCC2^+^ group (42.75 %) and highest in the GPx4^−^RCC2^-^ group (81.25 %). Statistical analysis revealed that the 5-year OS in the GPx4^+^RCC2^+^ group was significantly lower in GPx4^−^RCC2^-^ group (*P* < 0.001), and the 5-year OS in the GPx4^−^RCC2^+^ group was significantly lower than in the GPx4^−^RCC2^-^ group (*P* = 0.029), No significant differences in 5-year OS were observed between the GPx4^−^RCC2^-^ group and GPx4^+^RCC2^-^ group (*P* = 0.232), or between the GPx4^−^RCC2^+^ and GPx4^+^RCC2^+^ groups (*P* = 0.369,Fig. 9G).

## 4 Discussion

This study reveals that GPx4 is highly expressed in GC tissues and is closely associated with peritoneal metastasis and overall survival in GC patients. Moreover, targeting GPx4-induced ROS inhibits peritoneal metastasis by promoting RCC2 ubiquitination and degradation through phosphorylation of RCC2 by Aurora A.

GPx4 is a key antioxidant enzyme that plays a significant role in regulating ferroptosis. It utilizes glutathione (GSH) to protect liposomes and biological membranes from oxidative degradation [33,34]. Recent studies have shown that GPx4 prevents ferroptosis by eliminating intracellular peroxides and promoting cell survival [35]. Disruption of redox homeostasis, whether by GPx4 gene knockout or modulation of its activity with various inducers, has profound effects on cells. Multiple studies [[36], [37], [38]] have found that GPx4 is highly expressed in various common malignancies, suggesting its role as an oncogene. For instance, GPx4 overexpression can activate PTEN/PI3K/AKT signaling and promoting metastasis via transcriptionally silencing GRHL3 (Grainyhead-like 3) expression in hepatocellular carcinoma [39]. Additionally, Li et al. [40] found that GPx4 homeostasis plays a critical role in orchestrating ferroptosis and cancer immunotherapy. Our studies confirmed that GPx4 is significantly upregulated as GC progresses and is closely linked to tumor metastasis and overall survival in GC patients. In addition, GPx4 knockdown significantly suppressed GC metastasis both in *vitro* and *vivo*.

We explored the mechanism by which GPx4 regulates GC progression and metastasis. Proteomic analysis revealed that RCC2 was significantly downregulated following GPx4 silencing. RCC2 is a highly conserved oncogene that plays a crucial role in regulating mitosis [17]. Its involvement in cancer has gained increasing attention due to its association with metastasis, chemotherapy resistance, and poor prognosis [18]. RCC2 overexpression can induce changes in the microenvironment, including EMT and extracellular matrix deposition through JNK (c-JUN N-terminal kinase) activation, thereby promoting invasion and metastasis [21]. RCC2 can also upregulate and stabilize SOX2 (SRY-box transcription factor 2) expression by inhibiting ubiquitination-mediated proteasome degradation, thus facilitating the progression of esophageal cancer [41]. In addition, RCC2 enhances glucose metabolism through BACH1-dependent transcriptional upregulation of hexokinase II in glioma [42]. In our study, we found that GC cells with high RCC2 expression significantly enhanced cell invasion and migration reducing the effectiveness of the GPx4 inhibitor JKE-1674 in inhibiting these processes in GC cells. We also confirmed a significant correlation between RCC2 and GPx4 expression in GC tissues, with high co-expression being associated with poor prognosis. These findings suggest that targeting GPx4 may regulate the invasion and metastasis by modulating RCC2 expression.

Additionally, we explored the mechanism by which GPx4 regulates RCC2 degradation. As an essential enzyme for ROS clearance, GPx4 is crucial for maintaining cellular activity. ROS influence various cellular behaviors, such as signaling and cell death, and also modulate the tumor microenvironment [43]. Increased ROS levels, induced by different cancer-related signals, can activate the immune system and promote the anti-tumor immune responses [44,45]. ROS accumulation resulting from GPx4 downregulation has been shown to enhance anti-tumor immune response and reshape tumor microenvironment by reducing lactic acid [46]. Therefore, we hypothesized that GPx4 might regulate RCC2 expression through ROS levels. By treating GC cell lines with exogenous ROS and lipid ROS, we confirmed that ROS accumulation induced by targeting or knocking down GPx4 suppresses peritoneal metastasis by promoting RCC2 ubiquitination and degradation through Aurora A-induced phosphorylation of RCC2 both in *vitro* and *vivo*. Phosphorylation can influence protein ubiquitination by affecting E3 ligase recognition or the subcellular localization of substrates. This led us to investigate whether RCC2 phosphorylation affects its ubiquitination in response to ROS. Our study confirms that phosphorylation at T418 and ubiquitination at K377 are crucial for regulating RCC2 degradation in response to ROS.

## 5 Conclusion

In this study, we identified GPx4 as a novel target in GC, crucial for driving GC progression and metastasis. We found that ROS accumulation, resulting from either targeting or knocking down GPx4, suppresses peritoneal metastasis by promoting RCC2 ubiquitination through Aurora A. Furthermore, the T418 phosphorylation site and the K377 ubiquitination site are essential for regulating RCC2 ubiquitination and degradation in response to ROS. Our study not only affirm the role of GPx4 in GC progression but also highlight it as a promising therapeutic target.

## CRediT authorship contribution statement

**Can Hu:** Data curation, Formal analysis, Methodology, Project administration, Validation, Writing – original draft, Resources, Writing – review & editing. **Jingli Xu:** Data curation, Formal analysis, Methodology, Writing – original draft. **Yanqiang Zhang:** Data curation, Formal analysis, Methodology, Software, Writing – original draft. **Ruolan Zhang:** Data curation, Methodology, Software. **Siwei Pan:** Methodology. **Jiahui Chen:** Data curation, Methodology. **Yan Wang:** Methodology. **Qianyu Zhao:** Methodology. **Yuqi Wang:** Methodology. **Weiwei Zhu:** Methodology. **Mengxuan Cao:** Methodology. **Shengjie Zhang:** Methodology. **Dan Zu:** Methodology. **Zhiyuan Xu:** Conceptualization, Funding acquisition, Supervision, Writing – review & editing. **Ji Jing:** Conceptualization, Supervision, Validation, Writing – review & editing. **Xiangdong Cheng:** Conceptualization, Funding acquisition, Project administration, Supervision, Writing – review & editing.

## Data and materials availability

The raw data from the single-cell RNA sequencing reported in this paper have been deposited in GEO, and the accession number is GSE239676. The code used in this study is available from the corresponding author on reasonable request. Detailed information is available from the corresponding authors upon reasonable request.

## Funding

This study was supported by The National Key Research and Development Program of China (2021YFA0910100), Healthy Zhejiang One Million People Cohort (K-20230085), National Natural Science Foundation of China (82304946, 82473489, 82403546), Post-doctoral Innovative Talent Support Program (BX2023375), Medical Science and Technology Project of Zhejiang Province (WKJ-ZJ-2202, 2023KY073), Natural Science Foundation of Zhejiang Province (LR21H280001, LMS25H160006), China Postdoctoral Science Foundation (2023M733563), Science and Technology Projects of Zhejiang Province (2022KY684), and Program of Zhejiang Provincial TCM Sci-tech Plan (2022ZQ020).

## Declaration of competing interest

The authors declare that they have no known competing financial interests or personal relationships that could have appeared to influence the work reported in this paper.
