# Supplementary material for: Inhibition of glutathione peroxidase 4 suppresses gastric cancer peritoneal metastasis via regulation of RCC2 homeostasis
Source: Redox Biol. 2025 Jan 30;80:103519. doi: 10.1016/j.redox.2025.103519 (PMC11847474; doi:10.1016/j.redox.2025.103519)
Supplement: Multimedia component 1 [file mmc1.docx]

Supplementary Figures


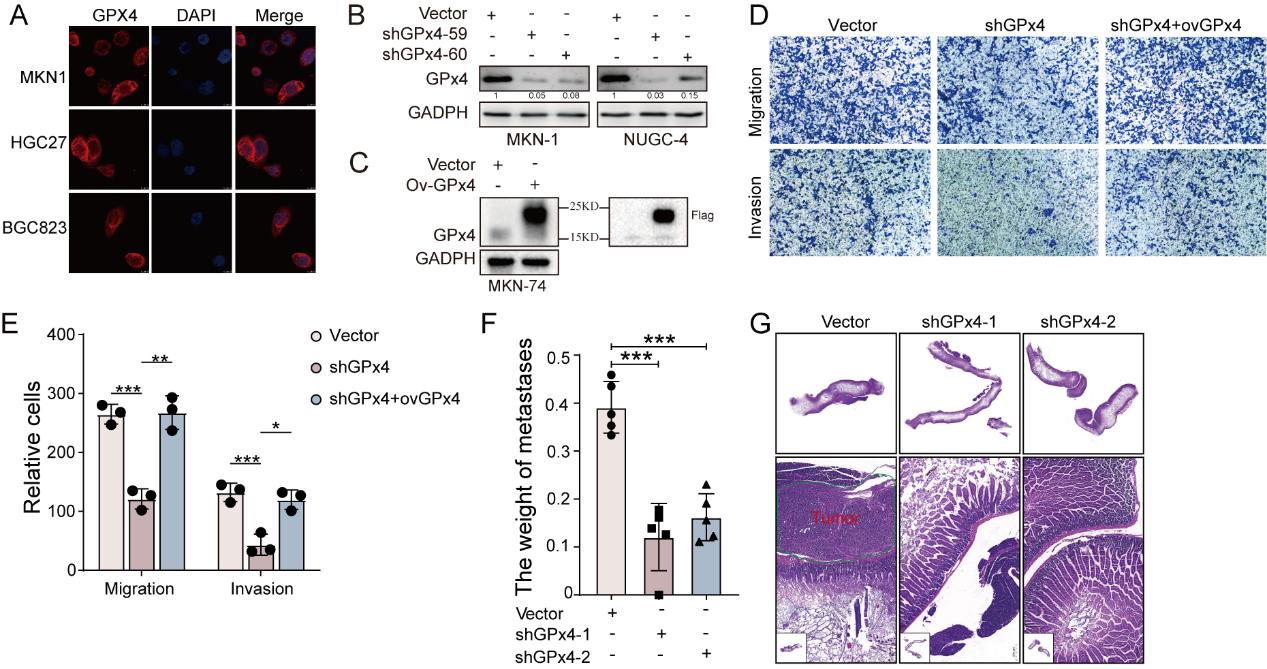


**Supplementary Figure 1. A.** The confocal shown that the localization of GPx4 in MKN1, HGC27 and BGC 823 GC cells. **B**. Western blot validation of GPx4 knockdown efficiency in MKN-1, NUGC-4 and MKN-45 GC cell lines following transfection with GPx4-shRNA and scrambled-shRNA. **C**. Western blot confirmation of GPx4 overexpression in MKN-74 GC and control. **D**. The transwell assay shown that the ability of invasion and migration could be rescued after overexpressing GPx4 in shGPx4 MKN1 GC cells. **E**. Quantitative analysis of transwell assay data. **F**. The total weight of peritoneal tumor in vector, shGPx4-1 and shGPx4-2 group. **G**. The representative images of H&E of intestine in vector, shGPx4-1 and shGPx4-2 group.


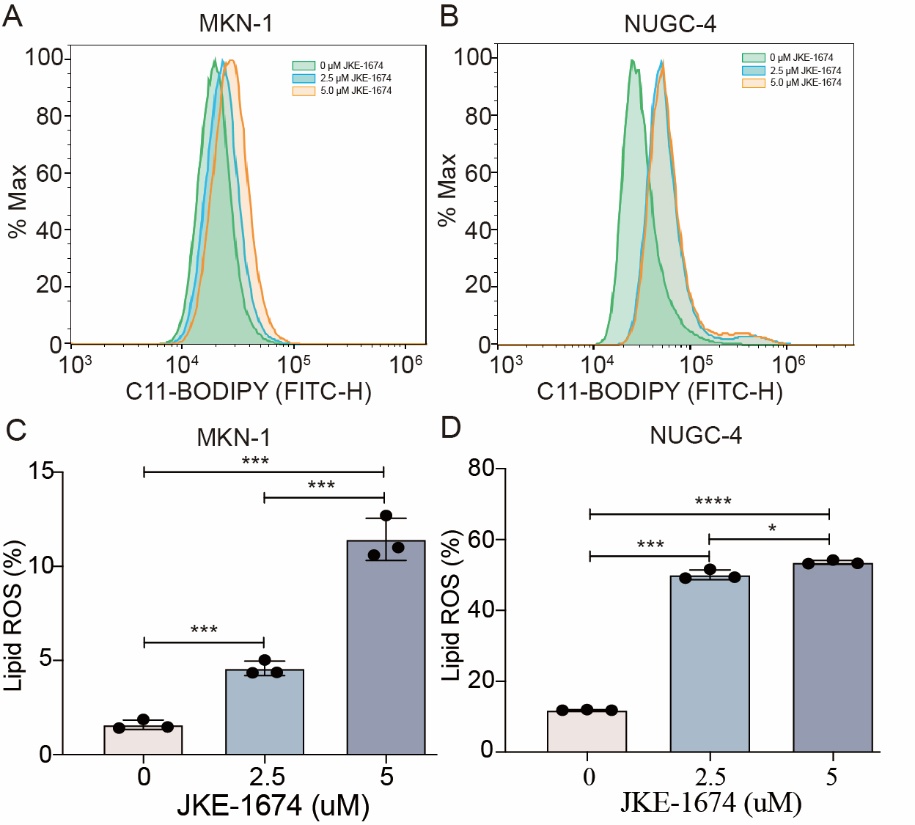


**Supplementary Figure 2. A-B**. Flow cytometry analysis of lipid ROS levels in MKN-1 and NUGC-4 GC cells treated with JEK-1674 (0, 2.5, 5.0 μM) for 24 hours. **C-D**. Quantitative data from the flow cytometry analysis of lipid ROS.


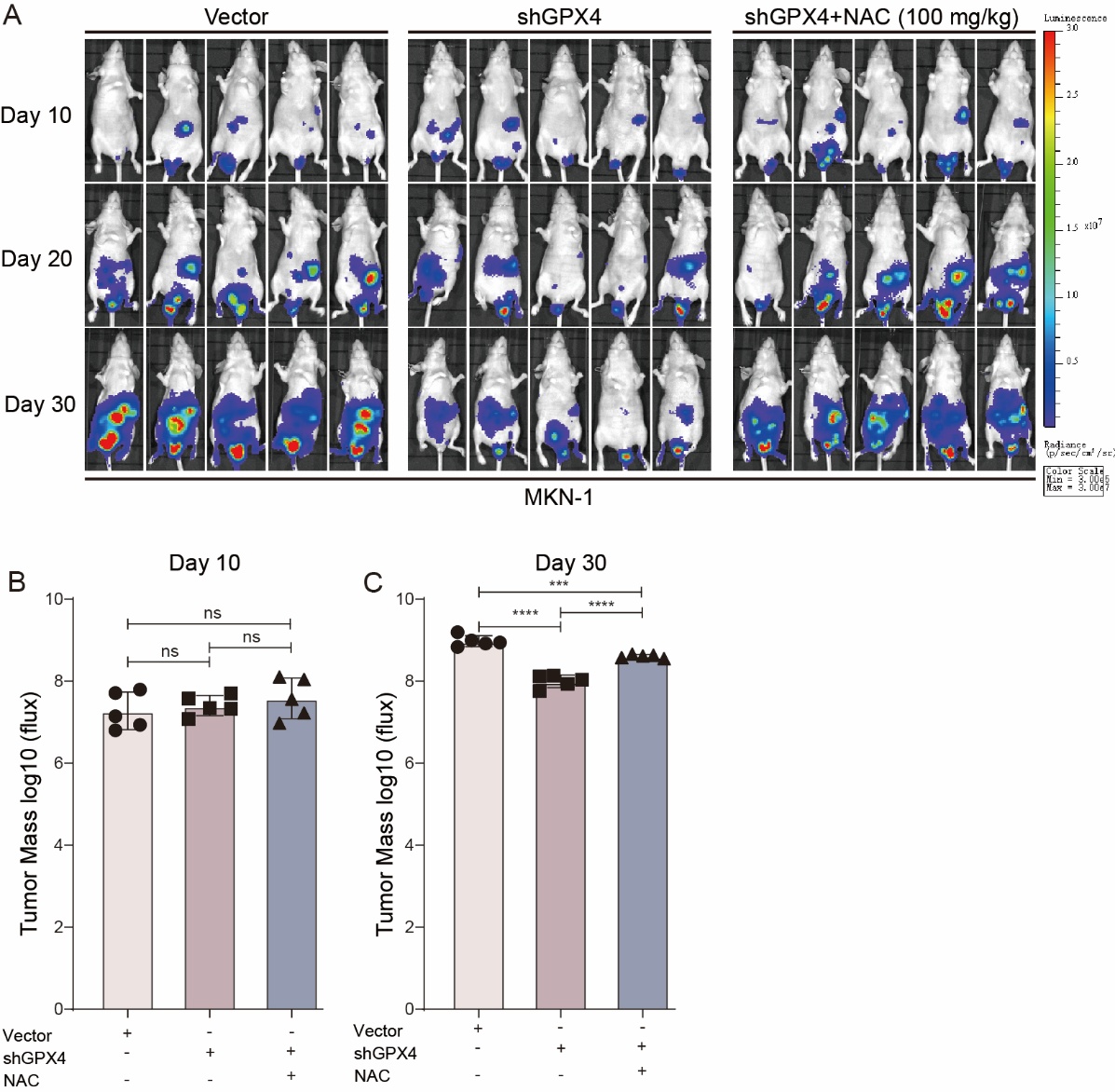


**Supplementary Figure 3. A**. The MKN-1 cells, at total of 6 × 10^6^ suspended in 200 µL saline solution, were administered intraperitoneally to male nude mice aged five to six weeks. For the functional validation, MKN-1 cells from the vector control group (Vector group), GPx4 knockdown group (shGPx4) were inoculated into the abdominal cavities of mice. The shGPx4 group was then randomly divided into two subgroups on day 10, which received either saline or NAC (100 mg/kg) via intraperitoneal injection twice a week for treatment (n = 5/each group). Luciferase signals were detected using an IVIS imaging system. **B**. The average tumor mass (determined by the detected photons/sec) of the mice in different group on day 10. **C**. The average tumor mass (determined by the detected photons/sec) of the mice in different group on day 30.


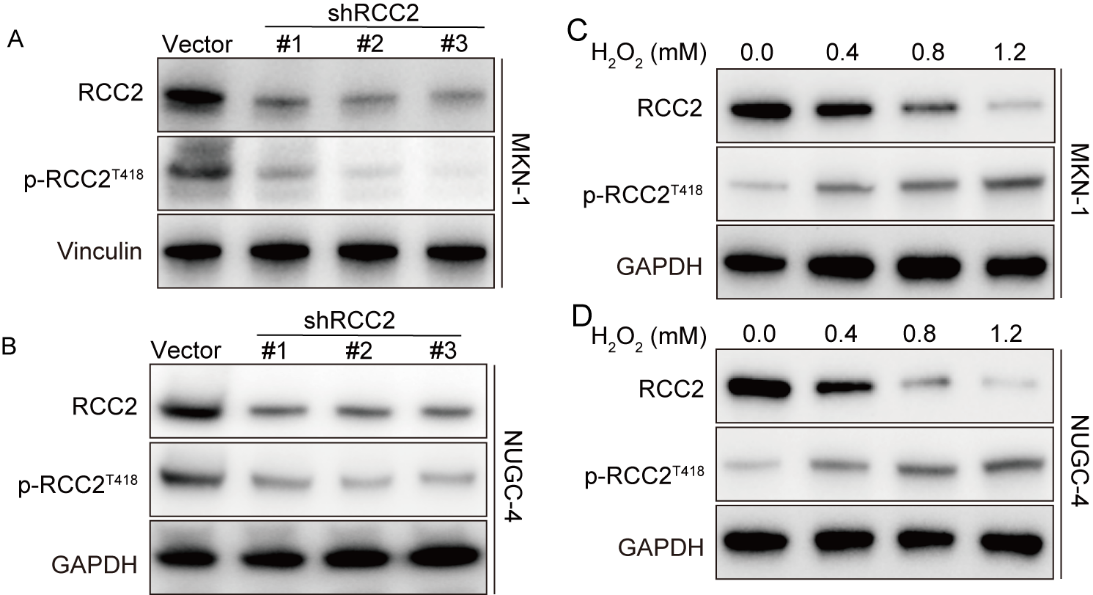


**Supplementary Figure 4. A-B**. Western blot analysis of RCC2 and phosphorylated-RCC2 at T418 (p-RCC2^T418^) expression in MKN-1 and NUGC-4 cells following RCC2 knockdown. **C-D**. Western blot analysis of RCC2 and phosphorylated-RCC2 at T418 (p-RCC2^T418^) expression in MKN-1 and NUGC-4 cells after treating with H_2_O_2_ (0.4 mM) for 6 hours.


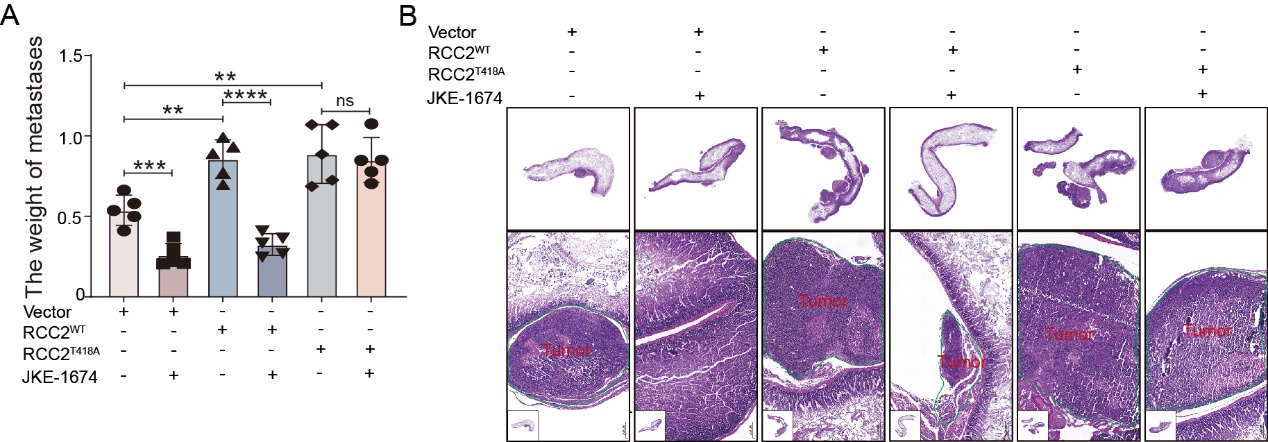


**Supplementary Figure 5. A**. The total weight of peritoneal tumor in n mice from different treatment groups. **B**. The representative images of H&E of intestine in n mice from different treatment groups.
